# Comparative safety of drug therapies used in hemophilia A and B in Canada: a multi-center, retrospective study

**DOI:** 10.1016/j.rpth.2025.103280

**Published:** 2025-12-02

**Authors:** Omotola O. Olasupo, Emma Iserman, Arun Keepanasseril, Quazi Ibrahim, Zainab Salim Ali Al-Housni, Federico Germini, Jean-Eric Tarride, Lawrence Mbuagbaw, Shannon Jackson, Ingrid Blydt-Hansen, Michelle Bech, Celina Woo, Mark Belletrutti, Alfonso Iorio, Davide Matino, Association of Hemophilia Clinic Directors of Canada (AHCDC), Roy Khalife, Kelsey Brose, Caroline Malcolmson, Kelsey Uminski, Ali Amid, Paul Moorehead, Jean St-Louis, Catherine Vezina, Jennifer Leung, Anna Serebrin, Marie-Claude Pelland-Marcotte, Soumitra Tole, Amye Harrigan, MacGregor Steele, Roona Sinha, Manuel Carcao, Anthony K.C. Chan, Chai W. Phua, Robert Klaassen, Man-Chiu Poon

**Affiliations:** 1 Department of Medicine, University of Ottawa, Canada; 2 The Ottawa Blood Disease Center, The Ottawa Hospital, Ottawa, Canada; 3 Ottawa Hospital Research Institute, Ottawa, Canada; 4 Saskatchewan Cancer Agency, Canada; 5 Division of Pediatric Hematology-Oncology, Department of Pediatric Medicine, The Hospital for Sick Children, University of Toronto, Canada; 6 Transfusion Medicine, Division of Hematopathology, Department of Pediatric Laboratory Medicine, The Hospital for Sick Children, University of Toronto, Canada; 7 Division of Hematology and Hematologic Malignancies, University of Calgary, Calgary, Alberta, Canada; 8 Division of Hematology, Oncology & BMT, Department of Pediatrics, Faculty of Medicine, University of British Columbia, Canada; 9 Pediatric Hematology, BC Children's Hospital, Vancouver, British Columbia, Canada; 10 Section of Pediatric Hematology/Oncology, Janeway Children’s Health and Rehabilitation Centre, St. John’s, Newfoundland, Newfoundland and Labrador, Canada; 11 Hématologie-Oncologie Hôpital Maisonneuve-Rosemont, Montreal, Québec, Canada; 12 Montreal Children's Hospital, Hôpital de Montréal pour enfants, Montreal, Québec, Canada; 13 Hematology, Kingston Health Sciences Center, Queen's University, Kingston, Ontario, Canada; 14 Department of Pediatrics, Faculty of Medicine and Dentistry, Edmonton Clinic Health Academy, Edmonton, Alberta, Canada; 15 Hémato-oncologue pédiatrique Centre Mère-Enfant Soleil, CHU de Québec, Québec, Canada; 16 Div. of Hematology/Oncology, Children's Hospital, London Health Sciences Centre, Western University, London, Ontario, Canada; 17 Division of Hematology, Central Zone, Nova Scotia Health, Nova Scotia, Ontario, Canada; 18 Alberta Children’s Hospital, University of Calgary, Calgary, Alberta, Canada; 19 Department of Pediatric Hematology/Oncology, University of Saskatchewan; 20 Department of Pediatrics, McMaster University, Hamilton, Ontario, Canada; 21 Paediatric Thrombosis and Hemostasis, McMaster Children’s Hospital - Hamilton Health Sciences, Hamilton, Ontario, Canada; 22 Schulich School of Medicine & Dentistry, Western University, London, Ontario, Canada; 23 Southwestern Ontario Bleeding Disorders Program, Adult Hematology, London Health Sciences Centre, London, Ontario, Canada; 24 Department of Pediatrics, University of Ottawa, Ottawa, Ontario, Canada; 25 Division of Hematology/Oncology, Children's Hospital of Eastern Ontario, Ottawa, Ontario, Canada; 26 Karpinski Klaassen Medicine Professional Corporation; 27 Division of Hematology and Hematologic Malignancies, Department of Medicine, University of Alberta, Alberta, Canada; 28 Southern Alberta Rare Blood and Bleeding Disorders Comprehensive Care Program, Foothills Hospital, Calgary, Alberta, Canada; 1 Department of Health Research Methods, Evidence, and Impact, McMaster University, Hamilton, Ontario, Canada; 2 Department of Medicine, McMaster University, Hamilton, Ontario, Canada; 3 Department of Biomedical Sciences, Clinical Epidemiology and Research Center, Humanitas University, Milan, Italy; 4 Centre for Health Economics and Policy Analysis, McMaster University, Hamilton, Ontario, Canada; 5 Biostatistics Unit, Father Sean O’Sullivan Research Centre, St Joseph’s Healthcare, Ontario, Canada; 6 Adult Bleeding Disorders Program, St. Paul’s Hospital, Vancouver, British Columbia, Canada; 7 Division of Hematology/Oncology/BMT, Department of Pediatrics, University of British Columbia, Vancouver, BC, Canada

**Keywords:** blood coagulation factors, drug safety, emicizumab, hemophilia, pharmacovigilance

## Abstract

**Background:**

Comparative safety data on hemophilia therapies are scarce.

**Objectives:**

To compare the risk of adverse drug reactions (ADRs) associated with extended-half-life (EHL) and standard-half-life (SHL) clotting factor therapies, bypassing agents, and emicizumab.

**Methods and Analysis:**

We analyzed Canadian Bleeding Disorders Registry data from 2018 to 2022. ADRs were defined as adverse events (AEs) if definitely, possibly, or probably treatment-related. We compared incidence rates of ADRs between therapies to estimate incidence rate ratios and 95% CIs.

**Results:**

We found a total of 183 AEs and 67 ADRs. Reported AEs varied from 6.1 to 14.8 events per 1000 patients per year. Allergic reactions were the most prevalent ADRs. A higher incidence of allergic reactions was associated with emicizumab compared with EHL (IRR 3.59; 95% CI, 1.43-9.00) and SHL (IRR 11.86; 95% CI, 4.73-29.72) clotting factor concentrates. Events reflecting inadequate hemostatic control and other unintended events occurred more often with emicizumab compared with SHL (IRR 6.39, 95% CI, 1.29-31.63) and EHL concentrates (IRR 2.77, 95% CI, 0.56-13.72). No inhibitor development was reported with emicizumab or bypassing agents. Cases of neurological events and thrombosis were reported when emicizumab was used in combination with other hemostatic therapies.

**Conclusion:**

This study highlights the relative safety of therapies approved for the management of hemophilia A and B. While more ADRs were reported with emicizumab, no inhibitor development was observed. However, novelty bias cannot be ruled out. Our estimates are limited by the use of routinely collected data with no adjustment for confounding due to low event rates and missing data.

## Essentials

• The comparative safety of hemophilia therapies used in Canada is yet to be fully assessed.
• Using registry data, we compared rates of adverse drug reactions for drug groups over 5 years.
• Allergic reactions were the most reported adverse drug reactions in the Canadian population.
• While more events were reported with emicizumab, we cannot rule out novelty bias.

## 1 Introduction

Clotting factor therapies, and more recently, the Factor VIII (FVIII) mimetic, emicizumab, used in hemophilia A, are standard of care therapies for treating and preventing bleeds in hemophilia [1]. Drug therapy options also include bypassing agents used in patients with neutralizing antibodies or inhibitors to clotting factor therapies and hemostatic agents such as desmopressin and tranexamic acid [2].

While pharmacological interventions provide therapeutic benefits, their use could also result in unintended adverse drug reactions (ADRs) [3,4].

An ADR is a response to a drug, which is noxious and unintended and that occurs at doses normally used for prophylaxis, diagnosis, therapy of disease, or the modification of physiologic function [5]. ADRs could be definitely, probably, or possibly related to the administered medication, based on the judgment at the time of reporting [6]. These are distinct from adverse events (AEs) which refer to any untoward medical occurrence that may present during treatment with a pharmaceutical product, but which does not necessarily have a causal relationship with this treatment [7,8].

With hemophilia being a rare disease, most studies are not sufficiently powered to assess the comparative safety of therapies used in its management. This motivated the creation of an active surveillance system, the European Haemophilia Safety Surveillance System (EUHASS), where adverse events (AEs) in 22,242 European patients have been prospectively monitored since 2008 [9].

From the EUHASS database inception until 2022, a total of 4228 AEs have been reported. These events include mortality, which account for 46.8% of all reports; malignancy (22%); inhibitor development (16.9%); thrombosis (8.4%); allergic reactions (5.8%); and transfusion-transmitted infections (0.02%) [10]. Assessment of these events based on EUHASS data have been described across different product formulations [9,11].

Adverse event reporting in the Canadian hemophilia population has historically been evaluated through the Canadian Hemophilia Surveillance System, the Canadian Hemophilia Assessment and Resource Management Information System, and more recently from 2015, through the Canadian Bleeding Disorders Registry (CBDR) [12]. Collecting the same adverse events as the EUHASS, the CBDR captures data from 23 Canadian hemophilia treatment centers (HTCs), where 3510 hemophilia A and 743 hemophilia B patients are currently being treated [12,13]. The availability of centralized data in the CBDR offers the opportunity to explore the real-world post-authorization safety of available therapies used in the Canadian population.

While ADRs for individual clotting factors, bypassing agents, and emicizumab have been previously described [[14], [15], [16]], we are not aware of any study evaluating the relative risk of ADRs associated with these therapies.

In this study, we assessed the comparative incidence of ADRs associated with hemostatic agents in the Canadian population with hemophilia A and B.

## 2 Methods

### 2.1 Study design and participants

This is a retrospective dynamic cohort study reported according to the Strengthening the Reporting of Observational Studies in Epidemiology checklist [17]. While the CBDR was created in 2015, full migration of data from the Canadian Hemophilia Surveillance System to the CBDR did not occur until 2018. Therefore, all patients with a diagnosis of hemophilia A or B receiving therapy at a Canadian HTC registered in the CBDR between 2018 and 2022 were eligible for inclusion. We also included patients with hemophilia A and B who received care at Canadian HTCs in the process of fully migrating records into the CBDR within the study period. We excluded patients diagnosed with acquired hemophilia and those diagnosed with other inherited bleeding disorders. At participating HTCs, AE data are proactively reported into the CBDR using standardized forms with drop down menus specifying data to be reported, including event description, products, and product lot numbers. Quarterly reports of AEs are tracked in the CBDR, with quarterly reminders sent to participating centers.

### 2.2 Ethical considerations

All data extracted and analyzed were deidentified according to personal health information protection principles for medical research involving human subjects [18]. This study was approved by the Hamilton Integrated Research Ethics Board in 2021 (CBDR/MyCBDR ID:13194-C), with an amendment for an updated dataset approved in 2023 (data cutoff date: 2022).

### 2.3 Population characteristics

We assessed demographic and clinical characteristics of the study population at the study entry (date of CBDR data entry), capturing data on age, weight, sex, hemophilia diagnosis (hemophilia A or B), hemophilia severity (mild, moderate, and severe), treatment plan (prophylaxis, secondary prophylaxis, on-demand therapy, and tolerization), and Haemophilia Joint Health Score.

### 2.4 Exposure

Study exposure was defined as any treatment with (i) standard-half-life (SHL) clotting factor concentrate, (ii) extended-half-life (EHL) clotting factor concentrate, (iii) bypassing agent, or (iv) emicizumab. Clotting factor concentrates are categorized based on pharmacokinetic profiles into SHL concentrates; with an average half-life of 8 to 12 hours for FVIII concentrates and 18 to 24 hours for factor IX (FIX) concentrates [[19], [20], [21]]. EHL concentrates have an average half-life of 18 to 22 hours for FVIII concentrates and 82 to 115 hours for EHL FIX concentrates [[19], [20], [21]]. We defined an exposure day as a calendar day in which a person with hemophilia was on treatment as recorded in the CBDR. The total exposure days is the total number of days in which patients were on treatment with a product. Available therapies and product categories patients were exposed to during the study period are provided in Supplementary Appendix 1.

### 2.5 Outcomes

We estimated the incidence of ADRs from AE reports with outcomes of interest defined as allergic or acute reactions, inhibitor development, poor effectiveness and other events, neurologic events, thrombosis, transfusion-transmitted infections, malignancy, and all-cause mortality.

Based on ADR definitions [5], we excluded events in which the relationship between the medication and the adverse event was determined to be unlikely or unrelated to the medication, or with missing data regarding treatment relationship. AE data extracted from the CBDR and identification of ADRs were adjudicated by members of the study team (OO, EI, ZA, and DM), who reviewed registry entries to confirm the relationship between the drug therapies and AE reports, based on temporality, biological plausibility, and documented information at the time of reporting. In cases where there was not enough information captured in the original report, we requested for more information from the reporting center.

Reported AEs and ADRs were defined according to standard EUHASS convention and World Federation of Hemophilia definitions [1,9].

Allergic or acute reactions were defined as immunologic responses to the administered medication, including but not limited to rash, pruritus, urticaria, swelling of the lips, and airways [22]. Inhibitors were defined as neutralizing alloantibodies to exogenous clotting FVIII or FIX, as detected by the Nijmegen-modified Bethesda assay [1]. Inhibitors are reported when detected at any titer above the laboratory normal limit (>0.6 Bethesda units [BU] for FVIII and ≥ 0.3 BU for FIX) on 2 separate occasions [9].

The outcome poor effectiveness and other events describes all bleeding-related events such as nosebleeds, continuous bleeding despite treatment, and other possible adverse events such as infections and complications with medical devices.

Neurologic events were events involving a disruption of blood flow to the brain, leading to damage and dysfunction of brain tissue, which includes hemorrhagic stroke, neuro-infections including meningitis, encephalitis, or myelitis, migraines, headaches, numbness or loss of touch sensation, and cognitive difficulties.

Thrombosis was defined as new episodes of myocardial infarction, ischemic stroke, pulmonary embolism, deep vein thrombosis, arterial thrombosis, or first-ever episodes of angina pectoris and transient ischemic attacks within or after 30-days of drug exposure [9].

Transfusion-transmitted infections include hepatitis B, hepatitis C, HIV, and other infections suspected to be transmitted by the clotting factor treatment [9].

### 2.6 Statistical analysis

Demographic and clinical characteristics of the population were described using summary statistics: mean, standard deviation (SD), median, minimum and maximum values for continuous variables, and frequencies and percentages for categorical variables. We estimated the incidence rates of ADRs for each year from the AE reports and compared incidence rates across therapy types to calculate incidence rate ratios (IRR) and incidence rate differences (IRD), with the corresponding 95% CIs [23]. None of the product groups was chosen as a fixed or standard reference, hence, we made pairwise comparisons across the drug categories. We assessed all patients with data in the CBDR for eligibility and did not estimate sample sizes a priori.

We explored the effects of potential confounders or effect modifiers such as treatment plan, hemophilia type (A or B), hemophilia severity, and age. Due to data availability and low event rates, we only conducted univariate exploratory analyses showing the distribution of ADRs based on these variables. Two-tailed *P* values of less than .05 were considered statistically significant. All analyses were conducted using Microsoft Excel for Microsoft 365 MSO (Version 2503 Build 16.0.18623.20116) and MedCalc Statistical Software version 23.2.1 (MedCalc Software Ltd; https://www.medcalc.org; 2025).

## 3 Results

### 3.1 Demographic and clinical characteristics

**Figure 1:**
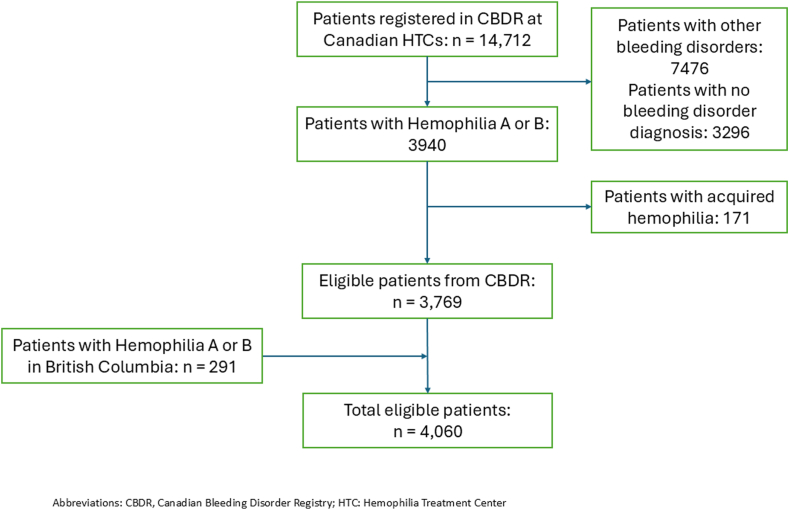
Study flow chart.

Our study cohort comprised of 4060 patients with a mean ± SD age of 34.7 ± 21.9 years. Most participants were male (95.7%), had hemophilia A (81.4%), and mild disease (51.9%). Of the 62.8% of the cohort who had treatment plans reported, the majority were on on-demand therapy (Table 1). A flowchart of study participants is provided in Figure 1.

**Table 1:** Demographic and clinical characteristics of the population.

Characteristics∗	Total 4060 (100%)	Hemophilia A 3337 (81.4%)	Hemophilia B 723 (18.6%)
Age, y			
Mean ± SD	34.7 ± 21.9	34.3 ± 21.9	36.7 ± 21.8
Median (min-max)	32.0 (0-99)	32.0 (0-99)	35.0 (0-94)
Age category; *n* (%)			
0-5	338 (8.3)	291 (8.7)	47 (6.5)
6-11	339 (8.3)	280 (8.4)	59 (8.2)
12-17	333 (8.2)	283 (8.5)	50 (6.9)
18-65	2622 (64.9)	2143 (64.2)	479 (66.3)
65+	428 (10.5)	340 (10.2)	88 (12.2)
Weight, kg			
*N*	2913	2366	547
Mean ± SD	70.9 ± 30.0	70.4 ± 30.1	73.2 ± 29.3
Median (min-max)	75.0 (1.0-205.0)	74.6 (2.0-205.0)	76.7 (1.0-172.0)
Sex; *n* (%)			
Male	3887 (95.7)	3207 (96.1)	680 (94.1)
Female	171 (4.2)	128 (3.8)	43 (5.9)
Unreported	2 (0.0)	2 (0.1)	0 (0.0)
Hemophilia severity; *n* (%)			
Mild	2107 (51.9)	1861 (55.8)	246 (34.0)
Moderate	590 (14.5)	332 (9.9)	258 (35.7)
Severe	1331 (32.8)	1121 (33.6)	210 (29.0)
Unreported	32 (0.8)	23 (0.7)	9 (1.3)
Treatment plan; *n* (%)			
Prophylaxis	1100 (27.1)	909 (27.2)	191 (26.4)
Secondary prophylaxis	22 (0.5)	20 (0.6)	2 (0.3)
On-demand	1410 (34.7)	1128 (33.8)	282 (39.0)
Tolerization	18 (0.4)	18 (0.5)	0 (0.0)
No plan reported	1510 (37.2)	1262 (37.8)	248 (34.3)
Haemophilia joint health score (HJHS)			
*N*	208	172	36
Mean ± SD	15.6 ± 17.4	15.2 ± 17.1	17.9 ± 18.8
Median (min-max)	10.0 (0.0-76.0)	10.0 (0.0-76.0)	10.5 (0.0-58.0)

∗ At study entry.

### 3.2 Adverse event (AE) reports

A total of 183 events were reported across the 5-year study period (2018-2022). AE reports varied from 8.2 events per 1000 patients in 2018 (the first study year), to 14.8 events per 1000 patients in 2022 (Table 2, Supplementary Appendix 2). The lowest event rate (6.1 events per 1000 patients) was reported in 2021, and the highest event rate (14.8 events per 1000 patients) was reported the following year, 2022.

**Table 2:** Adverse event (AE) reports in patients with Hemophilia (CBDR 2018-2022).

Adverse Events, *n* (events per 1000)	2018 *N* = 3555	2019 *N* = 3723	2020 *N* = 3836	2021 *N* = 3936	2022 *N* = 4060	Total *N* = 4060
COVID 19	0 (0.0)	0 (0.0)	8 (2.1)	5 (1.3)	28 (6.9)	41 (10.1)
Allergic or acute reaction	3 (0.8)	8 (2.2)	9 (2.3)	3 (0.8)	12 (3.0)	35 (8.6)
Inhibitor development	10 (2.8)	8 (2.1)	7 (1.8)	3 (0.8)	3 (0.7)	31 (7.6)
Death	6 (1.7)	5 (1.3)	8 (2.1)	5 (1.3)	4 (1.0)	28 (6.9)
Malignancy	6 (1.7)	7 (1.9)	1 (0.3)	1 (0.3)	7 (1.7)	22 (5.4)
Poor effectiveness/other events	3 (0.8)	4 (1.1)	5 (1.3)	3 (0.8)	3 (0.7)	18 (4.4)
Neurologic event	0 (0.0)	0 (0.0)	0 (0.0)	2 (0.5)	2 (0.5)	4 (1.0)
Thrombosis	1 (0.3)	0 (0.0)	0 (0.0)	2 (0.5)	1 (0.2)	4 (1.0)
Total number of adverse events, *n* (events per 1000)	29∗(8.2)	32 (8.6)	38 (9.9)	24 (6.1)	60 (14.8)	183 (45.1)

*n* = number of events; *N* = number of study participants.

∗ Includes 12 reports reported in the Canadian Hemophilia Assessment and Resource Management Information System (CHARMS)

### 3.3 Adverse drug reactions

**Figure 2:**
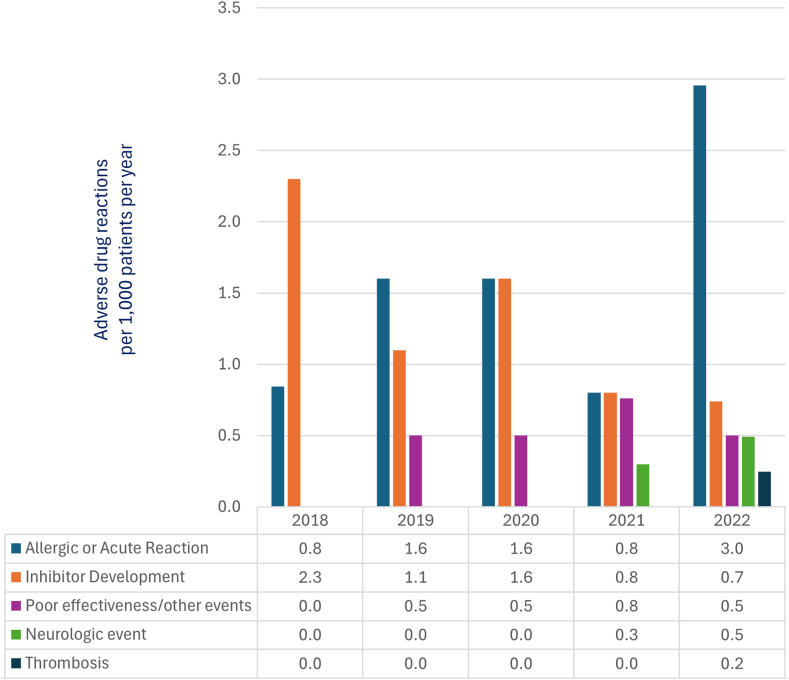
Adverse drug reactions per 1000 patients per year (CBDR 2018–2022).

Of the 183 AEs reported, 67 (36.6%) were treatment related: 23 definitely related, 29 possibly related, and 15 probably related (Supplementary Appendix 3). COVID-19 infections, deaths, and malignancies were determined as not associated with any of the therapies (Figure 2; Table 3).

**Table 3:** Adverse drug reactions (ADRs) in patients with hemophilia (CBDR 2018-2022).

Adverse drug reactions *n* (events per 1000)	2018 *N* = 3555	2019 *N* = 3723	2020 *N* = 3836	2021 *N* = 3936	2022 *N* = 4060	Total *N* = 4060
Allergic or acute reaction	3 (0.8)	6 (1.6)	6 (1.6)	3 (0.8)	12 (3.0)	30 (7.4)
Inhibitor development	8 (2.3)	4 (1.1)	6 (1.6)	3 (0.8)	3 (0.7)	24 (5.9)
Poor effectiveness/other events	0 (0.0)	2 (0.5)	2 (0.5)	3 (0.8)	2 (0.5)	9 (2.2)
Neurologic event	0 (0.0)	0 (0.0)	0 (0.0)	1 (0.3)	2 (0.5)	3 (0.7)
Thrombosis	0 (0.0)	0 (0.0)	0 (0.0)	0 (0.0)	1 (0.2)	1 (0.2)
Adverse drug reactions, *n* (events per 1000)	11 (3.1)	12 (3.2)	14 (3.6)	10 (2.5)	20 (4.9)	67 (16.4)

ADR incidence varied from a minimum of 2.5 events per 1000 patients in 2021 to a maximum of 4.9 events per 1000 patients in 2022.

Allergic or acute reactions had the highest period prevalence (*n* = 30) with reporting rates ranging from 0.8 to 3.0 events per 1000 patients per year (3 to 12 events per year). This is followed by inhibitor development (*n* = 24) and outcomes reflecting inadequate hemostatic control and other unintended events (*n* = 9).

No transfusion-transmitted infections, malignancy, or mortality were associated with the therapies examined.

### 3.4 Comparative incidence of ADRs

#### 3.4.1 Allergic or acute reactions to treatment products

Allergic reactions reported were predominantly rash [11], cardiopulmonary events (difficulty breathing or shortness of breath, tightness of chest, palpitation, tachycardia, chest pain), and injection site reactions [4]. Other allergic reactions included fever, malaise, lethargy, hives, urticaria, nausea and vomiting, anxiety, and dizziness.

Emicizumab was associated with 17 more allergic reaction reports per 1000 person years compared with SHL therapies (IRD 16.57; 95% CI, 11.74-21.40; *P* < .001), representing an 11.86-fold increase in allergic reactions reported compared with SHL (IRR 11.86; 95% CI, 4.73-29.72; *P* < .001), and a 3.59-fold increase compared with EHL therapies (IRR 3.59; 95% CI, 1.43-9.00; *P* = .006). Of note, allergic or acute reactions attributed to emicizumab were predominantly rash [7] and injection site reactions [4], which are expected and common skin-related outcomes [24].

Compared with SHL therapies, EHL therapies were associated with 3.3 times reporting for allergic reactions (IRR 3.30; 95% CI, 1.26-8.67; *P* = .015). No allergic reactions were reported with bypassing agents (Table 4, Supplementary Appendix 4).

**Table 4:** Comparative incidence–incidence rate ratio of ADRs (CBDR: 2018–2022).

Adverse drug reactions (ADR)	Total ADR *n* = 67	Product groups	ADR	Total exposure days	Incidence rate (per 1000 person years)	Incidence rate ratio (95% CI), *P*-value	Incidence rate ratio (95% CI), *P*-value
Allergic or acute reaction							
	30	SHL	7	1,673,949	1.53 (0.61-3.14)	(Ref)	0.30 (0.11-0.79), .015
		EHL	10	724,349	5.04 (2.42-9.27)	3.30 (1.26-10.22), .015	(Ref)
		Emicizumab	13	262,162	18.1 (9.64-30.95)	11.86 (4.73-29.72), < .001	3.59 (1.43-9.00), .006
		BPA	0	55,426	0	0	
Inhibitor development							
	24	SHL	22	1,673,949	4.8 (3.01-7.26)	(Ref)	4.80 (0.05-0.85), .030
		EHL	2	724,349	1.0 (0.12-3.64)	0.21 (0.05-0.85), .030	(Ref)
		BPA	0	55,426	0	0	0
		Emicizumab	0	262,162	0	0	0
Poor effectiveness or other event∗							
	9	SHL	3	1,673,949	0.65 (0.13-1.91)	(Ref)	0.43 (0.09-2.13), .305
		EHL	3	724,349	1.51 (0.31-4.42)	2.31 (0.47-11.45), .305	(Ref)
		Emicizumab	3	262,162	4.18 (0.86-12.21)	6.39 (1.29-31.63), .023	2.77 (0.56-13.72), .215
		BPA	0	55,426	0	0	
Neurological event							
	3	SHL	3∗∗	1,673,949	0.65 (0.13-1.91)	(Ref)	0.23 (0.04-1.41), .113
		Emicizumab	2∗∗	262,162	2.78 (0.34-10.06)	4.26 (0.71-25.47), .113	(Ref)
		EHL	0	724,349	0	0	0
		BPA	0	55,426	0	0	0
Thrombosis							
	1	BPA	1∗∗∗	55,426	6.59 (0.17-36.69)	(Ref)	4.76 (0.30-100), .296
		Emicizumab	1∗∗∗	262,162	1.39 (0.04-7.76)	0.21 (0.01-3.38), .272	(Ref)
		SHL	0	1,673,949	0	0	0
		EHL	0	724,349	0	0	0

ADR, adverse drug reaction; BPA, bypassing agent; EHL, extended-half-life clotting factor therapy; SHL, standard half-life clotting factor therapy.

∗ Poor effectiveness or other adverse drug reactions included unresolved bleeding (*n* = 4), nosebleeds (*n* = 1), hematoma (*n* = 2), line infection (*n* = 1), and tooth abscess (*n* = 1).

∗∗ Two cases of headaches were linked to a combination of emicizumab and simoctocog α (*n* = 1) and emicizumab and turoctocog α (*n* = 1).

∗∗∗ One thrombosis event was linked to a combination of emicizumab and eptacog α.

#### 3.4.2 Inhibitor development

More than half of inhibitors (13/24; 54.2%) were reported in children 0-5 years (Supplementary Appendix 5). The risk for developing inhibitors was lower with EHL compared to SHL therapies (IRR 0.21; 95% CI; 0.05-0.85; *P* = .030). No inhibitor development was reported with emicizumab and bypassing agents.

#### 3.4.3 Poor effectiveness and other events

Nine events were reported as poor effectiveness or other adverse drug reactions. These included unresolved bleeding (*n* = 4), nosebleeds (*n* = 1), hematoma (*n* = 2), line infection (*n* = 1), and tooth abscess (*n* = 1). The incidence of these events was higher with emicizumab compared with SHL therapies (IRR 6.39; 95% CI, 1.29-31.63; *P* = .023). The incidence rate for this category of events was not significantly different between EHL therapies and emicizumab (*P* = .215), or between EHL and SHL therapies (*P* = .305). No poor effectiveness or other adverse drug reactions were reported with bypassing agents.

#### 3.4.4 Neurologic events

Two events (headaches) were linked to a combination of emicizumab and simoctocog alfa (SHL; *n* = 1) and emicizumab and turoctocog alfa (SHL; *n* = 1). One case of hemorrhagic stroke was linked to nonacog alfa (SHL; *n* = 1). No neurologic event was associated with EHL or bypassing agents.

#### 3.4.5 Thrombosis

One report of thrombosis was associated with a combination of emicizumab and activated eptacog alfa (bypassing agent). Thrombosis was not reported for any of the other product categories.

#### 3.4.6 ADRs by age group, treatment plan, hemophilia type, and severity

Exploring the distribution of ADR by age groups, 45% of ADRs were reported in adults aged 18-65 years, who make up 64.9% of the cohort, followed by 33% reported in children 0-5 years (8.3% of the cohort) (Supplementary Appendix 5).

More events were reported in patients with hemophilia A compared to hemophilia B (82.1% vs 17.9%), when compared with the cohort distribution of 81.4% and 18.6% (Supplementary Appendix 6).

In patients with treatment plans reported at the time of AE reporting, a higher percentage of ADRs were reported in patients on prophylaxis regimens compared with those on on-demand therapy (16.4%: vs 7.5%) (Supplementary Appendix 7).

Fifty out of the 67 ADRs (74.6%) were reported in patients with severe hemophilia (32.8% of the cohort), compared with 13.4% for those with mild hemophilia (51.9% of the cohort), and 11.9% in those with moderate hemophilia (14.5% of the cohort) (Supplementary Appendix 8).

## 4 Discussion

In this study, we examined the comparative safety of drug therapies used in the management of hemophilia A and B in the Canadian population. We estimated and compared the risk of ADRs associated with 4 categories of drug therapies: EHL clotting factor therapies, SHL clotting factor therapies, bypassing agents, and emicizumab. Our study showed that the reporting of AEs following bleed prevention and treatment in hemophilia varied between 7 to 15 events per 1000 patients per year, while ADRs varied between 3 to 5 reports per 1000 patients per year.

Allergic reactions, inhibitor development, and events reflecting inadequate hemostatic control were the top 3 ADRs reported at Canadian HTCs. Compared with SHL and EHL clotting factor concentrates, more cases of allergic reactions and events reflecting inadequate hemostatic control were reported with emicizumab. More cases of inhibitor development were reported with SHL compared with EHL concentrates, and no inhibitor development was reported with emicizumab and bypassing agents. Neurologic events and thrombosis were rarely reported; hence, not enough data is available to assess the relative risk for these events across the product categories.

Our findings are consistent with findings from previous studies, which show that clotting factor replacement therapies have good safety profiles, with an overall rate of adverse events estimated to be 0.13% [25].

The development of inhibitors, an important AE associated with clotting factor replacement therapies [26], is estimated to occur at a rate 20% to 30% during the initial 50 exposure days in previously untreated patients with severe hemophilia A, and as low as one to 5 cases per 1000 patients per year in previously treated patients [27]. This is consistent with our findings, with incidence rates ranging from one to 3 (0.8-2.3) events per 1000 patients per year. Although we did not assess this incidence rate by exposure status (ie rates in previously treated patients vs previously untreated patients), we identified that inhibitors predominantly developed in pediatric patients, who were more likely to be previously untreated or minimally exposed to clotting factor concentrates.

There was a trend toward reduced inhibitor development, which corresponds to the timeline emicizumab was introduced into clinical practice as primary prophylaxis. This is consistent with reports from the EUHASS showing a reduction in inhibitor development from 25% before 2016, to 6% in 2022 in previously untreated patients with severe hemophilia A [28].

There were no reports of inhibitor development associated with bypassing agents or emicizumab in our study. This could be attributed to the fact that patients on bypassing agents already have inhibitors or a history of inhibitors, and those with no inhibitors who are being treated with emicizumab are likely not receiving clotting factor therapies routinely and therefore not regularly assessed for inhibitor development.

In systematic reviews examining the efficacy and safety of emicizumab [29,30], evidence from randomized controlled trials suggests that emicizumab prophylaxis likely increased total adverse events compared with on-demand clotting factor replacement in patients without inhibitors (RR 2.83, 95% CI, 1.47-5.47, *n* = 54) [31], and in those with inhibitors (RR 1.97, 95% CI, 1.26-3.10) [31]. The most frequent adverse event reported with emicizumab was injection site reactions [[27], [28], [29], [30]].

Compared with the other pharmacological interventions (clotting factor therapies and bypassing agents), emicizumab is a relatively new therapy—and being the only subcutaneously administered option newly introduced to clinical practice during our study period, patients are more likely to report AEs and are more likely to be reminded to report AEs at initiation compared to the other therapies. This heightened scrutiny results in selective recall, new drug effect, or novelty bias, which could lead to an overestimation of the risk of adverse effects [31].

The opposite also applies to SHL clotting factor concentrates, where patients are less likely to report events such as nosebleeds and hematomas. To mitigate this, an analysis of AE and ADR rates in the first 6-12 months following a product switch or a new user-cohort design would ensure that the incidence rate of ADRs can be estimated in relation to the exposure time. However, we do not have enough person-level exposure data in the current analysis, especially for the SHL and EHL clotting factors, which have been in use for much longer than the other product categories.

There were no bleeding-related adverse events or events reflecting poor effectiveness associated with bypassing agents in our study. This could be a case of underreporting of events due to the suboptimal or less reliable effectiveness, which is expected with bypassing agents compared with replacement therapies in patients without inhibitors. With no universally validated laboratory assays to monitor the efficacy of bypassing agents, the assessment of bleeding control is relatively more dependent on clinical judgment compared with other therapies, and inadequate hemostasis and other treatment-attributed events could be unreported [32].

Evidence from our study suggests that more ADRs were reported in patients on prophylaxis regimens compared with those on on-demand therapy, and more events were reported in patients with severe hemophilia compared to mild or moderate hemophilia. This could be a function of the biological gradient ie, a result of the number of doses received—with more drug doses used in severe vs mild disease and more doses used in prophylaxis compared with on-demand treatment [33].

### 4.1 Strengths and limitations of this study

Our study offers the advantage of looking at multiple exposures at the same time, as well as the opportunity to measure multiple safety outcomes for each exposure. Describing the occurrence of adverse events across different clotting factor formulations used in Canada provides a national overview of the safety of these medications. Hence, our findings are generalizable to the Canadian hemophilia population.

The integration of treatment records across Canadian HTCs in the CBDR, as well as possible linkage with the Web-Accessible Population Pharmacokinetic Service-Hemophilia [34], and the Patient Reported Outcomes, Burdens and Experiences database [35], provides clinicians, researchers, and interest holders in hemophilia care, an opportunity to improve patient-relevant outcomes through ongoing treatment evaluation and monitoring. However, despite being an active drug safety surveillance system, we estimate that there is underreporting of adverse events in the CBDR. Twelve AE reports captured in the Canadian Hemophilia Assessment and Resource Management Information System did not contain information on treatment relationships with drug exposure; hence, these were not included in our analysis of ADRs.

Event rates were low in this study; therefore, ADR incidence rate comparisons were not adjusted for differences in patients’ demographic and clinical characteristics such as age, hemophilia type, hemophilia severity, and treatment plan, which could introduce confounding. Also, being a non-interventional study using routinely collected data, which is not specifically designed for drug safety assessment, we cannot rule out confounding as well as selection bias.

Another limitation of our study is that, while we examined the treatment relationship of the AE reports with the drug exposure, we did not assess the severity (Grade I-IV; mild, moderate, severe, life-threatening, death) and seriousness (serious or non-serious) of reported the ADRs based on the ICH criteria [8]. Future studies should include more granular collection of drug safety data.

## 5 Conclusion

Our analysis examines the comparative safety of approved therapies for the prevention and treatment of bleeds in people with hemophilia A or B. While AEs and ADRs in hemophilia A and B are rare, these events are not nonexistent. Clinicians and patients should watch out for immunological reactions and poor effectiveness outcomes when being exposed to newer therapeutic agents. Treatment decisions should be optimized to fit the best safety and therapeutic profile of available medications.

## Appendix

Association of Hemophilia Clinic Directors of Canada (AHCDC) study group:

Roy Khalife^1,2,3^, Kelsey Brose^4^, Caroline Malcolmson^5,6^, Kelsey Uminski^7^, Ali Amid^8,9^, Paul Moorehead^10^, Jean St-Louis,^11^ Catherine Vezina^12^, Jennifer Leung^13^, Anna Serebrin^14^, Marie-Claude Pelland-Marcotte^15^, Soumitra Tole^16^, Amye Harrigan^17^, MacGregor Steele^18^, Roona Sinha^19^, Manuel Carcao^20^, Anthony K. C. Chan^20,21^, Chai W. Phua^22,23^, Robert Klaassen,^24,25,26^ Man-Chiu Poon,^27,28^

^1^ Department of Medicine, University of Ottawa, Ottawa, Ontario, Canada

^2^ The Ottawa Blood Disease Center, The Ottawa Hospital, Ottawa, Ontario, Canada

^3^ Ottawa Hospital Research Institute, Ottawa, Ontario, Canada

^4^ Saskatchewan Cancer Agency, Saskatchewan, Canada

^5^ Division of Pediatric Hematology-Oncology, Department of Pediatric Medicine, The Hospital for Sick Children, University of Toronto, Toronto, Ontario, Canada

^6^ Transfusion Medicine, Division of Hematopathology, Department of Pediatric Laboratory Medicine, The Hospital for Sick Children, University of Toronto, Toronto, Ontario, Canada

^7^ Division of Hematology and Hematologic Malignancies, University of Calgary, Calgary, Alberta, Canada

^8^ Division of Hematology, Oncology & BMT, Department of Pediatrics, Faculty of Medicine, University of British Columbia, Vancouver, British Columbia, Canada

^9^ Pediatric Hematology, BC Children's Hospital, Vancouver, British Columbia, Canada

^10^ Section of Pediatric Hematology/Oncology, Janeway Children’s Health and Rehabilitation Centre, St. John’s, Newfoundland, Newfoundland and Labrador, Canada

^11^ Hématologie-Oncologie Hôpital Maisonneuve-Rosemont, Montreal, Québec, Canada

^12^ Montreal Children's Hospital, Hôpital de Montréal pour enfants, Montreal, Québec, Canada

^23^ Hematology, Kingston Health Sciences Center, Queen's University, Kingston, Ontario, Canada

^14^ Department of Pediatrics, Faculty of Medicine and Dentistry, Edmonton Clinic Health Academy, Edmonton, Alberta, Canada

^15^ Hémato-oncologue pédiatrique Centre Mère-Enfant Soleil, CHU de Québec, Québec, Canada

^16^ Div. of Hematology/Oncology, Children's Hospital, London Health Sciences Centre, Western University, London, Ontario, Canada

^17^ Division of Hematology, Central Zone, Nova Scotia Health, Nova Scotia, Ontario, Canada

^18^ Alberta Children’s Hospital, University of Calgary, Calgary, Alberta, Canada.

^19^ Department of Pediatric Hematology/Oncology, University of Saskatchewan, Saskatoon, Saskatchewan, Canada

^20^ Department of Pediatrics, McMaster University, Hamilton, Ontario, Canada

^21^ Paediatric Thrombosis and Hemostasis, McMaster Children’s Hospital - Hamilton Health Sciences, Hamilton, Ontario, Canada

^22^ Schulich School of Medicine & Dentistry, Western University, London, Ontario, Canada

^23^ Southwestern Ontario Bleeding Disorders Program, Adult Hematology, London Health Sciences Centre, London, Ontario, Canada

^24^ Department of Pediatrics, University of Ottawa, Ottawa, Ontario, Canada

^25^ Division of Hematology/Oncology, Children's Hospital of Eastern Ontario, Ottawa, Ontario, Canada

^26^ Karpinski Klaassen Medicine Professional Corporation, Canada

^27^ Division of Hematology and Hematologic Malignancies, Department of Medicine, University of Alberta, Alberta, Canada

^28^ Southern Alberta Rare Blood and Bleeding Disorders Comprehensive Care Program, Foothills Hospital, Calgary, Alberta, Canada
