# Supplementary material for: Comparative safety of drug therapies used in hemophilia A and B in Canada: a multi-center, retrospective study
Source: Res Pract Thromb Haemost. 2025 Dec 2;10(1):103280. doi: 10.1016/j.rpth.2025.103280 (PMC12799776; doi:10.1016/j.rpth.2025.103280)
Supplement: Supplementary Appendix [file mmc1.docx]

**APPENDIX I – AHCDC study group**

Roy Khalife^8,9,10^, Kelsey Brose^11^, Caroline Malcolmson^12,13^, Kelsey Uminski^14^, Ali Amid^15,16^, Paul Moorehead,^17^ Jean St-Louis,^18^ Catherine Vezina^19^, Jennifer Leung^20^, Anna Serebrin^21^, Marie-Claude Pelland-Marcotte^22^, Soumitra Tole^23^, Amye Harrigan^24^, MacGregor Steele^25^, Roona Sinha^26^, Manuel Carcao^12^, Anthony K. C. Chan^27,28^, Chai W. Phua^29,30^, Robert Klaassen,^31,32,33^ Man-Chiu Poon,^34,35^

**Affiliations**

^8^ Department of Medicine, University of Ottawa

^9^ The Ottawa Blood Disease Center, The Ottawa Hospital, Ottawa

^10^ Ottawa Hospital Research Institute, Ottawa

^11^ Saskatchewan Cancer Agency

^12^ Division of Pediatric Hematology-Oncology, Department of Pediatric Medicine, The Hospital for Sick Children, University of Toronto

^13^ Transfusion Medicine, Division of Hematopathology, Department of Pediatric Laboratory Medicine, The Hospital for Sick Children, University of Toronto

^14^ Division of Hematology and Hematologic Malignancies, University of Calgary

^15^ Division of Hematology, Oncology & BMT, Department of Pediatrics, Faculty of Medicine, University of British Columbia

^16^ Pediatric Hematology, BC Children's Hospital, Vancouver, British Columbia

^17^ Section of Pediatric Hematology/Oncology, Janeway Children’s Health and Rehabilitation Centre, St. John’s, Newfoundland, Newfoundland and Labrador.

^18^ Hématologie-Oncologie Hôpital Maisonneuve-Rosemont, Montreal, QC

^19^ Montreal Children's Hospital, Hôpital de Montréal pour enfants, Montreal, QC

^20^ Hematology, Kingston Health Sciences Center, Queen's University, Kingston, ON

^21^ Department of Pediatrics, Faculty of Medicine and Dentistry, Edmonton Clinic Health Academy, Edmonton, AB

^22^ Hémato-oncologue pédiatrique Centre Mère-Enfant Soleil, CHU de Québec, Québec

^23^ Div. of Hematology/Oncology, Children's Hospital, London Health Sciences Centre, Western University, London, ON

^24^ Division of Hematology, Central Zone, Nova Scotia Health, Nova Scotia, Ontario

^25^ Alberta Children’s Hospital, University of Calgary, Calgary, Alberta.

^26^ Department of Pediatric Hematology/Oncology, University of Saskatchewan.

^27^ Department of Pediatrics, McMaster University, Hamilton, ON

^28^ Paediatric Thrombosis and Hemostasis, McMaster Children’s Hospital - Hamilton Health Sciences, Hamilton, ON

^29^ Schulich School of Medicine & Dentistry, Western University, London, ON

^30^ Southwestern Ontario Bleeding Disorders Program, Adult Hematology, London Health Sciences Centre, London, ON

^31^ Department of Pediatrics, University of Ottawa, Ottawa, ON

^32^ Division of Hematology/Oncology, Children's Hospital of Eastern Ontario, Ottawa, ON

^33^ Karpinski Klaassen Medicine Professional Corporation

^34^ Division of Hematology and Hematologic Malignancies, Department of Medicine, University of Alberta

^35^ Southern Alberta Rare Blood and Bleeding Disorders Comprehensive Care Program

Foothills Hospital, Calgary, Alberta

**APPENDIX II: Therapies used in hemophilia A and B (CBDR: 2018 - 2022)**

| **SN** | **Clotting Factor Product** | **Description** | **Half-life (hrs)*** | **Manufacturer** |
| --- | --- | --- | --- | --- |
| **Factor VIII Deficiency (Hemophilia A)** | | |  |  |
| **SHL, Plasma-derived** | |  |  |  |
| 1 | Humate-P | Antihemophilic Factor/von Willebrand Factor Complex | FVIII: 12.2.  vWF:Rco: 11 | CSL Behring |
| 2 | Wilate | Human von Willebrand Factor/Coagulation Factor VIII Complex | 15 | Octapharma |
|  | **SHL, Recombinant** | **Description** | **Half-life (hrs)** | **Manufacturer** |
| 3 | octocog alfa  (Advate ^®^) | Full-length rFVIII, no functional VWF: no addition of human- or animal-derived plasma proteins or albumin in the cell culture, purification, or final formulation. | 11.98 | Shire/Takeda |
| 4 | octocog alfa  (Kovaltry ^®^) | Recombinant FVIII. Manufacture includes a detergent-based, viral inactivation step as well as a 20nm viral filtration step. | 13.8 | Bayer |
| 5 | Antihemophilic factor  (Helixate FS ^®^) | Full-length rFVIII; no VWF.  Produced in a baby hamster kidney (BHK) cell line. Formulated with sucrose. | 12 | Bayer - CSL Behring |
| 6 | octocog alfa (bhk)  (Kogenate FS ^®^) | Full-length rFVIII; no VWF. (FS: Formulated with sucrose). Albumin not added during purification or formulation. | 11 to 15 | Bayer |
| 7 | simoctocog alfa  (Nuwiq ^®^) | B-domain deleted, no VWF. SD treatment and nanofiltration. 9500 IU/mg (no albumin added) | 12.5 to 17.1 | Octapharma |
| 8 | Antihaemophilic factor [Recombinant Coagulation Factor VIII (rhc)  (Recombinate ^®)^ | Full length rFVIII, no functional VWF. Human serum albumin added as stabilizer | 15 | Novo Nordisk |
| 9 | antihemophilic factor  (Xyntha ^®)^ | B domain deleted rFVIII, no VWF. No monoclonal antibodies, human, or animal protein added to the cell culture, or used in purification or final formulation. | 14.8 | Pfizer |
| 10 | turoctocog alfa  (Zonovate ^®^) | B-domain deleted, no VWF.  Cell lines: Chinese hamster ovary (CHO) | 7.65 to 10.69 | Novo Nordisk |
|  | **EHL, Recombinant** | **Description** | **Half-life (hrs)** | **Manufacturer** |
| 11 | rurioctocog alfa pegol  (Adynovate ^®^) | Recombinant FVIII conjugate with 20 kDa polyethylene glycol (PEGylated rFVIII) | 13.43 to 14.69 | Shire/Takeda |
| 12 | efmoroctocog alfa  (Eloctate ^®^) | Recombinant fusion protein (B-domain deleted FVIII and dimeric Fc component of human IgG1), no VWF. Produced in a human embryonic kidney (HEK) cell line. | 19 | Biogen-Idec |
| 13 | Turoctocog alfa pegol  Esperoct ^®^ (N8-GP) | antihemophilic factor (recombinant) glycoPEGylated | 17 to 22 | Novo Nordisk |
| **Factor IX Deficiency (Hemophilia B)** | | |  |  |
|  | **SHL, Plasma-derived** | **Description** | **Half-life (hrs.)** | **Manufacturer** |
| 14 | Immunine VH | Freeze dried (human) Factor IX concentrate of high purity containing traces of Factors II, VII and X (<0.02 IU /. 1 IU factor IX) | 17.4 | Shire - Takeda |
| 15 | Mononine | Extraneous plasma-derived proteins, including Factors II, VII, and X, purified by use of immunoaffinity chromatography. | 22.6 | CSL Behring |
|  | **SHL, Recombinant** | **Description** | **Half-life (hrs.)** | **Manufacturer** |
| 16 | nonacog alfa  (BeneFIX ^®^) | Full-length rFIX. No monoclonal antibodies, human or animal protein added to the cell culture or used in purification or final formulation. | 18.8 | Pfizer |
| 17 | Nonacog gamma  (Rixubis ^®^) | Recombinant Coagulation Factor IX (rFIX), Nonacog gamma for Injection | 26.7 | Takeda |
|  | **EHL, Recombinant** | **Description** | **Half-life (hrs.)** | **Manufacturer** |
| 18 | eftrenonacog alfa  (Alprolix ^®^) | Human coagulation factor IX, Fc fusion protein (rFIXFc). Produced by recombinant DNA technology in a human embryonic kidney (HEK) cell line without the addition of any exogenous human- or animal-derived protein in the cell culture, purification, or final formulation. | 82.1 | Sobi, Bioverativ |
| 19 | Coagulation Factor IX (Recombinant), Pegylated  (Rebinyn ^®^) | Recombinant FIX conjugate with 40 kDa polyethylene glycol (PEGylated rFIX) | 13-17yrs: 103. >/=18yrs: 115 | Novo Nordisk |
|  | **Bypassing Agents** | **Description** | **Half-life (hrs.)** | **Manufacturer** |
| 20 | Anti-Inhibitor Coagulant Complex  (FEIBA VH and FEIBA-NF ^®^) | Plasma-derived (pd) Activated Prothrombin Complex Concentrate (aPCC). No heparin added; FII, FVII, FIX, and FX in a relatively balanced ratio. Vapor heated (VH). | 6 to 12 | Shire |
| 21 | eptacog alfa, activated  (NiaStase and NiaStase RT) | Activated recombinant human blood coagulation Factor VII (rFVIIa). Room temperature (RT) | 2.9 (Non-bleeding state) 2.3 (Bleeding State) | Novo Nordisk |
| **Monoclonal antibody** | | **Description** | **Therapeutic class** | **Manufacturer** |
| 1 | Emicizumab  (Hemlibra ^®^) | Humanized bispecific monoclonal antibody produced using genetically engineered Chinese hamster ovary (CHO) cells | Monoclonal antibody | Roche |
| * Half-live estimates were obtained from product monographs. | | | | |

**APPENDIX III: Adverse event reports per 1,000 patients per year (CBDR 2018 – 2022)**

**APPENDIX IV: Adverse drug reactions and relationship with drug exposure**

| ADR | 2018 | 2019 | 2020 | 2021 | 2022 | Total |
| --- | --- | --- | --- | --- | --- | --- |
|  | 3555 | 3723 | 3836 | 3936 | 4060 | 4060 |
| Allergic or acute reactions    Definitely related  Possibly related  Probably related | 3 (0.8)  0 (0.0)  3 (0.8)  0 (0.0) | 6 (1.6)  2 (0.5)  2 (0.5)  2 (0.5) | 6 (1.6)  3 (0.8)  3 (0.8)  0 (0.0) | 3 (0.8)  0 (0.0)  2 (0.5)  1 (0.3) | 12 (3.0)  9 (2.2)  3 (0.7)  0 (0.0) | 30 (7.4)  14 (3.4)  13 (3.2)  3 (0.7) |
| Inhibitor development    Definitely related  Possibly related  Probably related | 8 (2.3)  2 (0.6)  2 (0.6)  4 (1.1) | 4 (1.1)  2 (0.5)  0 (0.0)  2 (0.5) | 6 (1.6)  1 (0.3)  4 (1.0)  1 (0.3) | 3 (0.8)  1 (0.3)  1 (0.3)  1 (0.3) | 3 (0.7)  1 (0.2)  1 (0.2)  1 (0.2) | 24 (5.9)  7 (1.7)  8 (2.0)  9 (2.2) |
| Poor Efficacy/Other  Definitely related  Possibly related  Probably related | 0 (0.0)  0 (0.0)  0 (0.0)  0 (0.0) | 2 (0.5)  0 (0.0)  2 (0.5)  0 (0.0) | 2 (0.5)  0 (0.0)  1 (0.3)  1 (0.3) | 3 (0.8)  1 (0.3)  1 (0.3)  1 (0.3) | 2 (0.5)  0 (0.0)  2 (0.5)  0 (0.0) | 9 (2.2)  1 (0.2)  6 (1.5)  2 (0.5) |
| Neurologic Event    Definitely related  Possibly related  Probably related | 0 (0.0)  0 (0.0)  0 (0.0)  0 (0.0) | 0 (0.0)  0 (0.0)  0 (0.0)  0 (0.0) | 0 (0.0)  0 (0.0)  0 (0.0)  0 (0.0) | 1 (0.3)  0 (0.0)  0 (0.0)  1 (0.3) | 2 (0.5)  1 (0.2)  1 (0.2)  0 (0.0) | 3 (0.7)  1 (0.2)  1 (0.2)  1 (0.2) |
| Thrombosis    Definitely related  Possibly related  Probably related | 0 (0.0)  0 (0.0)  0 (0.0)  0 (0.0) | 0 (0.0)  0 (0.0)  0 (0.0)  0 (0.0) | 0 (0.0)  0 (0.0)  0 (0.0)  0 (0.0) | 0 (0.0)  0 (0.0)  0 (0.0)  0 (0.0) | 1 (0.2)  0 (0.0)  1 (0.2)  0 (0.0) | 1 (0.2)  0 (0.0)  1 (0.2)  0 (0.0) |
| ADRs, n (n per ‘000)    Definitely related  Possibly related  Probably related | 11 (3.1)  2 (0.6)  5 (1.4)  4 (1.1) | 12 (3.2)  4 (1.1)  4 (1.1)  4 (1.1) | 14 (3.6)  4 (1.0)  8 (2.1)  2 (0.5) | 10 (2.5)  2 (0.5)  4 (1.0)  4 (1.0) | 20 (4.9)  11 (2.7)  8 (2.0)  1 (0.2) | 67 (16.4)  23 (5.7)  29 (7.1)  15 (3.7) |

**APPENDIX V: Incidence rate differences**

| **Adverse drug reactions (ADR)** | **Total ADR n = 67** | **Product groups** | **ADR** | **Total exposure days** | **Incidence rate (per 1,000 person years)** | **Incidence rate difference**  **(95% CI), p-value** | **Incidence rate difference**  **(95% CI), p-value** |
| --- | --- | --- | --- | --- | --- | --- | --- |
| **Allergic or Acute Reaction** | | | | | | | |
|  | 30 | SHL | 7 | 1,673,949 | 1.53 (0.61 -3.14) | (Ref) | -3.51 (-6.19, -0.834), 0.010 |
|  |  | EHL | 10 | 724,349 | 5.04 (2.42-9.27) | 3.51 (0.83, 6.19), 0.010 | (Ref) |
|  |  | Emicizumab | 13 | 262,162 | 18.1 (9.64-30.95) | 16.57 (11.74, 21.40), <.001 | 13.06 (5.19, 20.93), 0.001 |
|  |  | BPA | 0 | 55,426 | 0 | 0 |  |
| Inhibitor Development | | | | | | | |
|  | 24 | SHL | 22 | 1,673,949 | 4.8 (3.00-7.26) | (Ref) | 3.79 (0.61, 6.97), 0.020 |
|  |  | EHL | 2 | 724,349 | 1.0 (0.12-3.64) | -3.79 (-6.97, -0.61),0.020 | (Ref) |
|  |  | BPA | 0 | 55,426 | 0 | 0 | 0 |
|  |  | Emicizumab | 0 | 262,162 | 0 | 0 | 0 |
| Poor Effectiveness or Other event | | | | | | | |
|  | 9 | SHL | 3 | 1,673,949 | 0.65 (0.13 – 1.91) | (Ref) | -0.86 (-2.45, 0.73), 0.291 |
|  |  | EHL | 3 | 724,349 | 1.51 (0.31 – 4.42) | 0.86 (-0.73, 2.45), 0.291 | (Ref) |
|  |  | Emicizumab | 3 | 262,162 | 4.18 (0.86 – 12.21) | 3.52 (0.88, 6.17), 0.009 | 2.67 (-1.36, 6.69), 0.194 |
|  |  | BPA | 0 | 55,426 | 0 | 0 |  |
| Neurological event | | | | | | | |
|  | 3 | SHL | 3* | 1,673,949 | 0.65 (0.13, 1.91) | (Ref) | -2.13 (-4.55, 0.28), 0.084 |
|  |  | Emicizumab | 2* | 262,162 | 2.78 (0.34, 10.06) | 2.13 (-0.28, 4.54), 0.084 | (Ref) |
|  |  | EHL | 0 | 724,349 | 0 | 0 | 0 |
|  |  | BPA | 0 | 55,426 | 0 | 0 | 0 |
| Thrombosis | | | | | | | |
|  | 1 | BPA | 1** | 55,426 | 6.59 (0.17, 36.69) | (Ref) | 5.19 (-3.19, 13.59), 0.225 |
|  |  | Emicizumab | 1** | 262,162 | 1.39 (0.04, 7.76) | -5.19 (-13.59, 3.20), 0.211 | (Ref) |
|  |  | SHL | 0 | 1,673,949 | 0 | 0 | 0 |
|  |  | EHL | 0 | 724,349 | 0 | 0 | 0 |
| Abbreviations: ADR, adverse drug reaction; BPA, bypassing agent; EHL, extended-half-life clotting factor therapy; SHL, standard half-life clotting factor therapy. | | | | | | | |
| *Cases of headaches were linked to a combination of emicizumab and simoctocog alfa (n=1) and emicizumab and turoctocog alfa (n=1). | | | | | | | |
| **One thrombosis event was linked to a combination of emicizumab and eptacog alfa. | | | | | | | |

**APPENDIX VI: ADR by age group**

|  | **0-5years** | | **6-11 years** | | **12-17** | | **18-65** | | **65+** | | **Total** |
| --- | --- | --- | --- | --- | --- | --- | --- | --- | --- | --- | --- |
| **ADR** | **No. of events** | **% of total** | **No. of events** | **% of total** | **No. of events** | **% of total** | **No. of events** | **% of total** | **No. of events** | **% of total** |  |
| **Allergic or Acute Reaction** | **6** | 20% | **2** | 7% | **3** | 10% | **18** | 60% | **1** | 3% | **30** |
| EHL | 5 | 56% |  | 0% | 1 | 11% | 3 | 33% | - | 0% | 9 |
| HEM | - | 0% | 2 | 15% | 1 | 8% | 9 | 69% | 1 | 8% | 13 |
| SHL | 1 | 13% |  | 0% | 1 | 13% | 6 | 75% | - | 0% | 8 |
| **Inhibitor Development** | **13** | 54% | **2** | 8% | **2** | 8% | **6** | 25% | **1** | 4% | **24** |
| EHL | 1 | 50% |  | 0% | 1 | 50% | - | 0% | - | 0% | 2 |
| SHL | 12 | 55% | 2 | 9% | 1 | 5% | 6 | 27% | 1 | 5% | 22 |
| **Neurological Event** | **-** | 0% | **-** | 0% | **-** | 0% | **3** | 100% | **-** | 0% | **3** |
| HEM + SHL | - | 0% | - | 0% | - | 0% | 2 | 100% | - | 0% | 2 |
| SHL | - | 0% | - | 0% | - | 0% | 1 | 100% | - | 0% | 1 |
| **Poor Efficacy/Other Event** | **3** | 33% | **1** | 11% | **1** | 11% | **2** | 22% | **2** | 22% | **9** |
| EHL | 1 | 33% | 1 | 33% | - | 0% | 1 | 33% | - | 0% | 3 |
| HEM | - | 0% | - | 0% | 1 | 33% | 1 | 33% | 1 | 33% | 3 |
| SHL | 2 | 67% | - | 0% | - | 0% | - | 0% | 1 | 33% | 3 |
| **Thrombosis** | **-** | 0% | **-** | 0% | **-** | 0% | **1** | 100% | **-** | 0% | **1** |
| HEM + BPA | - | 0% | - | 0% | - | 0% | 1 | 100% | - | 0% | 1 |
| **Grand Total** | **22** | 33% | **5** | 7% | **6** | 9% | **30** | 45% | **4** | 6% | **67** |
| Abbreviations: BPA, bypassing agents; EHL, extended half-life clotting factor concentrates; HEM, emicizumab; SHL, standard half-life clotting factor concentrates | | | | | | | | | | | |

**APPENDIX VII: ADR by hemophilia type**

|  | **Hemophilia A** | | **Hemophilia B** | | **Total** |
| --- | --- | --- | --- | --- | --- |
| **ADR** | **No. of events** | **% of total** | **No. of events** | **% of total** |  |
| **Allergic or Acute Reaction** | **22** | 73% | **8** | 27% | **30** |
| EHL | 3 | 33% | 6 | 67% | 9 |
| HEM | 13 | 100% | - | 0% | 13 |
| SHL | 6 | 75% | 2 | 25% | 8 |
| **Inhibitor Development** | **23** | 96% | **1** | 4% | **24** |
| EHL | 1 | 50% | 1 | 50% | 2 |
| SHL | 22 | 100% | - | 0% | 22 |
| **Neurological Event** | **2** | 67% | **1** | 33% | **3** |
| HEM + SHL | 2 | 100% | - | 0% | 2 |
| SHL | - | 0% | 1 | 100% | 1 |
| **Poor Efficacy/Other Event** | **7** | 78% | **2** | 22% | **9** |
| EHL | 1 | 33% | 2 | 67% | 3 |
| HEM | 3 | 100% | - | 0% | 3 |
| SHL | 3 | 100% | - | 0% | 3 |
| **Thrombosis** | **1** | 100% | **-** | 0% | **1** |
| HEM + BPA | 1 | 100% | - | 0% | 1 |
| **Grand Total** | **55** | 82% | **12** | 18% | **67** |
| Abbreviations: BPA, bypassing agents; EHL, extended half-life clotting factor concentrates; HEM, emicizumab; SHL, standard half-life clotting factor concentrates | | | | | |

**APPENDIX VIII: ADR by treatment plan reported at time of adverse event report**

|  | **ON-DEMAND** | | | | **PROPHYLAXIS** | | | |  | **PLAN UNREPORTED** | |  |
| --- | --- | --- | --- | --- | --- | --- | --- | --- | --- | --- | --- | --- |
| **ADR** | **No. of events** | **% of total** | **Total Exposure days** | **IR** | **No. of events** | **% of total** | **Total Exposure days** | **IR** | **IRR** | **No. of events** | **% of total** | **Total** |
| **Allergic or Acute Reaction** | **1** | 3% |  |  | **7** | 23% |  |  |  | **22** | 73% | **30** |
| EHL | 1 | 11% | 98,619 | 3.70 | 2 | 22% | 625,730 | 1.17 | (Ref) | 6 | 67% | 9 |
| HEM | - | 0% | - | - | 5 | 38% | 262,162 | 6.96 | 5.97 (0.98, 62.66) | 8 | 62% | 13 |
| SHL | - | 0% | 335,618 | - | - | 0% | 1,338,331 | - |  | 8 | 100% | 8 |
| **Inhibitor Development** | **4** | 17% |  |  | **1** | 4% |  |  |  | **19** | 79% | **24** |
| EHL | - | 0% | 98,619 | - | - | 0% | 625,730 | - |  | 2 | 100% | 2 |
| SHL | 4 | 18% | 335,618 | 4.35 | 1 | 5% | 1,338,331 | 0.27 |  | 17 | 77% | 22 |
| **Neurological Event** | **-** | 0% |  | **-** | **-** | 0% |  | **-** |  | **3** | 100% | **3** |
| HEM + SHL | - | 0% | 335,618 | - | - | 0% | 1,600,493 | - |  | 2 | 100% | 2 |
| SHL | - | 0% | 335,618 | - | - | 0% | 1,338,331 | - |  | 1 | 100% | 1 |
| **Poor Efficacy/Other Event** | **-** | 0% |  | **-** | **3** | 33% |  |  |  | **6** | 67% | **9** |
| EHL | - | 0% | 98,619 | - | 1 | 33% | 625,730 | 0.58 | (Ref) | 2 | 67% | 3 |
| HEM | - | 0% | - |  | 2 | 67% | 262,162 | 2.78 | 4.77 (0.25, 281.93) | 1 | 33% | 3 |
| SHL | - | 0% | 335,618 | **-** | **-** | 0% | 1,338,331 | - |  | 3 | 100% | 3 |
| **Thrombosis** | - | 0% |  | **-** | **-** | 0% |  | - |  | **1** | 100% | **1** |
| HEM + BPA | - | 0% | 29,069 | **-** | - | 0% | 288,519 | - |  | 1 | 100% | 1 |
| **Total** | **5** | 7% |  |  | **11** | 16% |  |  |  | **51** | 76% | **67** |
| Abbreviations: BPA, bypassing agents; EHL, extended half-life clotting factor concentrates; HEM, emicizumab; SHL, standard half-life clotting factor concentrates | | | | | | | | | | | | |

**APPENDIX IX: ADR by hemophilia severity**

|  | **MILD HEMOPHILIA** | | **MODERATE HEMOPHILIA** | | **SEVERE HEMOPHILIA** | | **Total** |
| --- | --- | --- | --- | --- | --- | --- | --- |
| **ADR** | **No. of events** | **% of total** | **No. of events** | **% of total** | **No. of events** | **% of total** |  |
| **Allergic or Acute Reaction** | **4** | 13% | **3** | 10% | **23** | 77% | **30** |
| EHL | 1 | 11% | - | 0% | 8 | 89% | 9 |
| HEM | - | 0% | 3 | 23% | 10 | 77% | 13 |
| SHL | 3 | 38% | - | 0% | 5 | 63% | 8 |
| **Inhibitor Development** | **4** | 17% | **3** | 13% | **17** | 71% | **24** |
| EHL | - | 0% | - | 0% | 2 | 100% | 2 |
| SHL | 4 | 18% | 3 | 14% | 15 | 68% | 22 |
| **Neurological Event** | - | 0% | **1** | 33% | **2** | 67% | **3** |
| HEM + SHL | - | 0% | - | 0% | 2 | 100% | 2 |
| SHL | - | 0% | 1 | 100% | - | 0% | 1 |
| **Poor Efficacy/Other Event** | **1** | 11% | **1** | 11% | **7** | 78% | **9** |
| EHL | - | 0% | - | 0% | 3 | 100% | 3 |
| HEM | - | 0% | 1 | 33% | 2 | 67% | 3 |
| SHL | 1 | 33% | - | 0% | 2 | 67% | 3 |
| **Thrombosis** | - | 0% | - | 0% | **1** | 100% | **1** |
| HEM + BPA | - | 0% | - | 0% | 1 | 100% | 1 |
| **Total** | **9** | **13%** | **8** | 12% | **50** | 75% | **67** |
| Abbreviations: BPA, bypassing agents; EHL, extended half-life clotting factor concentrates; HEM, emicizumab; SHL, standard half-life clotting factor concentrates | | | | | | | |
